# Acoustic Monitoring of Professionally Managed Marine Mammals for Health and Welfare Insights

**DOI:** 10.3390/ani13132124

**Published:** 2023-06-27

**Authors:** Kelley A. Winship, Brittany L. Jones

**Affiliations:** National Marine Mammal Foundation, 2240 Shelter Island Dr., Suite 200, San Diego, CA 92106, USA

**Keywords:** marine mammal welfare, acoustic welfare, anthropogenic sound

## Abstract

**Simple Summary:**

Marine mammal welfare research of professionally managed species has primarily focused on enrichment, habitat usage, and activity, as well as the impacts of human-oriented training sessions. However, the importance of sound in the welfare of marine mammals has rarely been mentioned. In this review, methods for acoustic welfare monitoring are discussed, including hearing tests, the incorporation of listening systems to monitor noise and communication, and cataloguing vocalizations in various health contexts. Examples from the US Navy Marine Mammal program are provided, as well as opportunities for facilities to initiate acoustic welfare monitoring. Suggested future directions of study, such as research examining the impact of sound on cognition, are also discussed.

**Abstract:**

Research evaluating marine mammal welfare and opportunities for advancements in the care of species housed in a professional facility have rapidly increased in the past decade. While topics, such as comfortable housing, adequate social opportunities, stimulating enrichment, and a high standard of medical care, have continued to receive attention from managers and scientists, there is a lack of established acoustic consideration for monitoring the welfare of these animals. Marine mammals rely on sound production and reception for navigation and communication. Regulations governing anthropogenic sound production in our oceans have been put in place by many countries around the world, largely based on the results of research with managed and trained animals, due to the potential negative impacts that unrestricted noise can have on marine mammals. However, there has not been an established best practice for the acoustic welfare monitoring of marine mammals in professional care. By monitoring animal hearing and vocal behavior, a more holistic view of animal welfare can be achieved through the early detection of anthropogenic sound sources, the acoustic behavior of the animals, and even the features of the calls. In this review, the practice of monitoring cetacean acoustic welfare through behavioral hearing tests and auditory evoked potentials (AEPs), passive acoustic monitoring, such as the Welfare Acoustic Monitoring System (WAMS), as well as ideas for using advanced technologies for utilizing vocal biomarkers of health are introduced and reviewed as opportunities for integration into marine mammal welfare plans.

## 1. Introduction

In the past decade, research investigating animal welfare of species living in professional care at zoological facilities has increased rapidly [1,2,3,4]. While farm animal welfare has long been a topic for legislators and farmers, more thorough investigations of the welfare of species in zoological facilities has been welcomed by researchers, animal care professionals, and the general public. Ethical concerns, such as the animals being free from prolonged fear and pain and being able to function well using their natural capabilities, have motivated many of the welfare models that have been adopted [5]. Widely accepted as the current welfare framework, the Five Domains [6] expanded upon the original Five Freedoms [7] and provided a more systematic means to evaluate welfare for human-managed animals [8]. The domains continue to be updated following advances in welfare research, such as a shift in focus from solely negative affective experiences [6] to the inclusion of positive experiences in the measure of welfare [8,9]. Currently, the domains consist of nutrition, physical environment, health, behavioral interactions, and mental states, with the impacts of human interactions included [10].

Marine mammal welfare, specifically in cetaceans, has become a focus of recent studies that aimed to establish measures to evaluate and improve the welfare of dolphins in professional care [11,12,13,14,15,16,17]. The Cetacean Welfare Assessment (C-Well^©^) established welfare measures based on the four welfare principles of good feeding, good housing, good health, and appropriate behavior [18]. Within the 36 measures used in C-Well^©^, neither acoustic environment nor acoustic behavior beyond the ability of dolphins to echolocate in more complex environments were included. While an environment that encourages the use of echolocation would be an important feature for odontocetes in terms of good housing, the necessity of an acoustically appropriate environment (i.e., sufficiently low levels of anthropogenic noise) was not considered [18]. Additionally, acoustic welfare (i.e., an appropriate acoustic environment and the ability to express natural acoustic-related behavior, such as communication) has not been mentioned in other reviews of marine mammal welfare [19], despite the importance of the species’ natural capabilities [5] relating to sound production and processing, which certainly impact welfare.

While the importance of acoustic welfare was discussed in 2015 [20], only a recent review of cetacean acoustic welfare has reinvigorated the conversation of incorporating acoustic information when evaluating the welfare of cetacean species [21]. This review echoes the call from Stevens et al. [21] and takes the current review one step further to focus on how acoustic welfare monitoring has been implemented, primarily at the US Navy’s Marine Mammal Program (MMP), provides an overview of recent technological advancements in applied tools for acoustic monitoring of cetaceans in human care, and outlines some proposed next steps for continued advancement in this area (see Table 1).

## 2. Monitoring Anthropogenic Sound Levels

The impact of sound on terrestrial mammals has received attention from zoos, with research evaluating the impact of anthropogenic noise relating to guest traffic [22,23,24,25], events, such as concerts [26], as well as periods of construction [27,28,29] on various species. The focus on noise impacts at zoos has resulted in facilities taking steps to mitigate noise during periods of construction for the sake of welfare [30], as well as testing the impact of signage to encourage quieter behavior from guests [25]. Despite the consistent interest in anthropogenic and environmental sounds in terrestrial mammals, there is comparatively little research examining the acoustic soundscapes of marine mammals in aquaria. There are likely multiple reasons for this oversight, such as the lack of awareness of sound sources that are occurring underwater compared to those that are audible to humans in air, the sensitivity of toothed whales to ultrasound, and the additional efforts it takes to record and monitor these sounds. Regardless of the environment in which marine mammals are housed (e.g., sea pens or closed system facilities), animals are exposed to anthropogenic sound sources, for example, from shipping traffic (see [31]) or from life support systems [32,33]. Therefore, facilities housing marine mammals ideally should monitor the level of sound the animals are exposed to [21] so that proper mitigation measures can be put in place or anomalies rectified [31]. Given the dependance cetaceans in particular have on sound production and reception for both navigation and communication, this is an area of welfare that deserves more attention [21].

### 2.1. Anthropogenic Sound in the Wild

The ocean is filled with biological, geologic, and anthropogenic sounds that can impact wild marine mammal welfare and alter health and behavior (see [34] for review). The rapid rise in vessel noise has become of increasing concern related to the management of wild marine mammals (see [35] for a review). The reliance on shipping as a primary means of transporting goods globally has resulted in higher noise levels in more areas of the ocean, and researchers have modeled the expansion of these operations as they expand into new environments [36]. As the noise level in the oceans has increased, some regulations have arisen to protect the marine environment.

The acoustic welfare of wild marine mammals, in terms of minimizing the negative impact of noise on health, has received attention from governmental agencies globally and has resulted in legislation and regulations aimed at minimizing the impact of sound on wild populations (e.g., the Marine Mammal Protection Act of 1972 in the United States (U.S.)). In the U.S., commercial and military activity is required to follow these regulations, and any instances in which regulations will not be followed (e.g., military exercises) must be approved by U.S. governmental agencies. Naval exercises have established guidance regarding impacts to marine mammals (see [37] for review). The existence of guidelines highlights the interest in the acoustic welfare of marine mammals from both scientific and ethical standpoints. In 2021, the National Marine Fisheries Service (NMFS) instituted new regulations in the Gulf of Mexico that require visual and acoustic observations for marine mammal detection, as well as other limits placed on the proximity of the equipment and vessels to the animals and the amount of time the vessels are allowed in areas of impact [38]. Much of the understanding of marine mammal hearing and the impacts of noise are the results of efforts of professional facilities investigating the topic, and further research will continue to result in meaningful changes in the monitoring and mitigation of noise for at-risk wild populations. 

### 2.2. Impacts of Anthropogenic Sound on Marine Mammals

Hearing thresholds are the lowest perceptible sounds an organism can hear based on the minimum sound pressure level (SPL) required to stimulate an auditory response. Exposure to intense noise sources can result in physical injury and/or impairment of hearing in the form of threshold shifts, which can be either permanent (PTS) or temporary (TTS). TTS is a noise-induced threshold shift (NITS) that eventually returns to normal. Understanding the levels at which TTS and PTS occur is crucial to ensuring health, welfare, safety, and other possible noise impacts on species. The initial studies of TTS in marine mammals took place at facilities in the U.S., sponsored in large part by the Office of Naval Research [39]. For a review of marine mammal TTS studies conducted from 1996 to 2015 and a thorough discussion of the testing parameters when evaluating TTS, see [39]. These studies, in conjunction with observations of the detrimental impacts of intense sounds in the marine environment, have resulted in regulations in the U.S., Canada, and the European Union, as well as the involvement of regional organizations in monitoring oceanic noise [40]. The evaluation of damage and subsequent welfare impacts has been a focal point of regulations, as establishing the threshold at which such sound results in harassment or injury is the basis for many of the laws guiding military and commercial sound exposure [41]. Auditory weighting functions to determine at which levels sound will become injurious to marine mammals have been formulated, though these are based on limited information from a handful of marine mammal species (see [42] for review). Exposure thresholds for TTS and PTS based on the type of noise exposure have been proposed [43,44] and are updated as new information is acquired [44,45]. While additional research regarding marine mammal hearing will continue to form the basis of future regulations protecting wild populations, this knowledge should also result in a more comprehensive welfare monitoring framework for animals in professionally managed settings.

### 2.3. Suggested Welfare Monitoring Measure: Annual Sound Level Recordings

**Figure 1:**
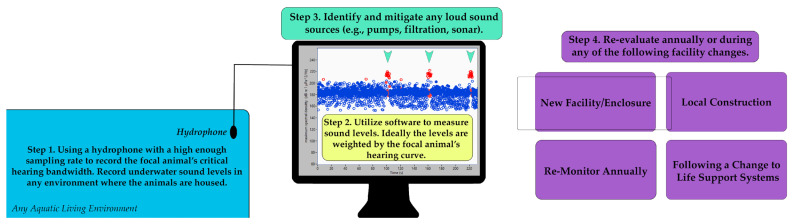
Workflow and proposed best practices for anthropogenic sound level monitoring of marine mammal aquatic living environments.

Because underwater noise sources are typically inaudible to humans in air, oftentimes, they end up going undetected and, therefore, unmitigated. Human hearing only extends up to about 20 kHz, whereas bottlenose dolphins (*Tursiops truncatus*) have excellent hearing and can potentially be affected by sound sources up to approximately 140 kHz [46]. Comments from critics of professional management of marine mammals have often focused on the acoustic environment of the habitats as being inadequate or harmful [47]. A recent collaborative effort was undertaken to examine the presence of underwater noise at 14 marine mammal facilities in the U.S., Bermuda, and Singapore [31]. Acoustic measurements in both land-based pools and natural seawater enclosures found that the risk of noise masking of animal communication was low. The experts found that noise levels in these enclosures were typically low-level and unlikely to cause concerns for the masking of echolocation or communication signals. However, infrequent and, in all cases, correctable noise levels that could be mitigated were identified. The authors recommended periodic sound level measurements as best practice for animal welfare [31]. In an evaluation of activity budgets of bottlenose dolphins, time periods in which construction noise was present resulted in behavioral changes, such as significantly faster swimming as well as decreased performance in training sessions [14]. Recently, an assessment of the anthropause due to COVID-19 found that dolphins that had become habituated to the anthropogenic sounds related to marine activity (e.g., jetskis and cruise ships) prior to the anthropause were more sensitive to playbacks following the reintroduction of human recreational activity near their lagoon [48]. At the MMP, an around-the-clock hydrophone array records the soundscape of the San Diego Bay where dolphins are housed in natural sea water environments [49] and “weights” the sound by the mid-frequency (MF)-cetacean hearing curve [44]. The user can set mid-frequency and high-frequency thresholds that, when exceeded, will trigger an alert to the stakeholder [49]. The possibility of TTS, and potentially PTS, is present at all facilities, regardless of structure. For example, sea pens can be subjected to boat traffic and sonar of recreational and military vessels, while closed facilities may experience elevated noise levels during periods of construction or malfunctioning life support systems generating abnormal noises. We echo Houser and colleagues’ [31] suggestion that facilities home to marine mammals should conduct periodic underwater sound-recording sessions to mitigate any sources that could be of detriment to the animals housed there (Figure 1). Periods of change, such as new construction or integration of new life support systems, should similarly ensure that sound levels have not significantly changed. 

## 3. Behavioral Hearing Tests and Auditory Evoked Potentials 

One aspect that we believe falls under the topic of acoustic monitoring of managed marine mammals is establishing the hearing threshold records of individuals [50,51,52,53,54]. Behavioral hearing tests have been successfully trained in bottlenose dolphins, (e.g., *Tursiops* sp. [55]; killer whales (*Orcinus orca,* [56,57]); false killer whales (*Pseudorca crassidens,* [58]); Pacific white-sided dolphins (*Lagenorhynchus obliquidens*, [59]); Tucuxis (*Sotalia fluviatilis*, [60]); harbor porpoises (*Phocoena phocoena,* [61]); California sea lions (*Zalophus californianus,* [62,63]); Stellar sea lions (*Eumetopias jubatus*, [64,65]); harbor seals (*Phoca vitulina,* [62,63]); Harp seals (*Pagophilus groenlandicus,* [66]); spotted seals (*Phoca largha*, [63]); ringed seals (*Pusa hispida*, [67]); bearded seals (*Erignathus barbatus;* [68]); northern elephant seals (*Mirounga angustirostris*, [62]); northern fur seals (*Callorhinus ursinus*, [69]); Hawaiian monk seals (*Neomonachus schauinslandi,* [70]); Pacific walrus (*Odobenus rosmarus divergens*, [71]); belugas (*Delphinapterus leucas*, [72]); Florida manatees (*Trichechus manatus latirostris*, [73]); polar bears (*Ursus maritimus*, [74]); and sea otters (*Enhydra lutris*, [75]). Behavioral hearing tests are the “gold-standard” of tests to determine individual animal sensitivity and range. Animals can be trained to voluntarily participate in sessions using different psychophysical procedures, such as a go/no-go paradigm, or a two-alternative forced choice (2AFC) design. For a go/no-go test, the animal is trained to signal the detection of a sound by touching a paddle or target [55,63,73], vocalizing [53,72], or producing another behavior (e.g., bubble-emitting vocalization; [56]). In instances of no sound detection, the animal remains in the listening position until cued to receive a reward. In a 2AFC task, the animal is presented with two stimuli and must make a choice. 2AFC tasks are arguably more challenging to train, given the paucity of studies that incorporate this strategy for hearing threshold research in marine mammals. However, this paradigm is often used to study echolocation [76,77]. Behavioral hearing tests result in not only data acquisition but also the opportunity for animals to be cognitively stimulated through the learning and testing process. While training such a behavior (particularly a go/no-go paradigm) through operant conditioning can be easily accomplished in most settings if the time and resources are dedicated to conceptual training, there may be situations in which animal hearing tests are more urgently needed or required in contexts in which training is not possible (e.g., rehabilitation cases).

As marine mammals age, hearing deteriorates [51,53,78,79]. Additionally, diminished hearing ability was noted as a factor in unsuccessful outcomes following release in rehabilitated cetaceans [80]. Following researcher suggestions in the assessment of rehabilitation cases [80,81], government regulations in the U.S. established that cetaceans showing severely compromised hearing are unsuitable for release [82]. Because these animals are not able to undergo training for behavioral hearing tests, the use of auditory evoked potentials (AEPs) emerged as a rapid and non-invasive way to monitor the electrical responses of an animal’s brain to auditory stimuli. Rather than train the animals to behaviorally respond to auditory cues, the process of training for AEP participation in dolphins simply requires training the animals to accept wearing surface electrodes and remain stationary while sounds are played either through a jawphone (i.e., a transducer embedded in a suction cup) or through an underwater sound projector, or the tests can be performed while a dolphin is out of water during a physical exam. Because AEPs are a more rapid means to collect hearing data compared to behavioral tests, this is the method that is primarily used with Navy dolphins. For pinnipeds, however, AEP tests have involved anesthesia and the use of subdermal needle electrodes in order to obtain accurate measurements [79].

During these AEP tests, stimuli at different frequencies are played, and the sound pressure level (SPL) is lowered until detection of an AEP is no longer achieved. By placing a jawphone on the dolphin’s lower jaw and playing stimuli, such as clicks or sinusoidal amplitude-modulated (SAM) tones, the audiogram (i.e., a plot of the hearing thresholds across frequencies) shape is estimated both in air and underwater [83]. Auditory steady-state response (ASSR) measurements are gathered by presenting stimuli at a fast enough rate that transient AEPs form overlapping signals, generating a quasi-steady-state response [84,85]. SAM tones are commonly used in ASSR measurements and have shown a strong correlation with behavioral results, albeit the AEP hearing thresholds generally underestimate (are higher than) behavioral thresholds to some degree [46,51,58,86]. Statistical relationships between click-evoked auditory brainstem responses (ABRs) and ASSRs have allowed for ABRs to serve as a more rapid means of hearing screening in dolphins in very time-limited situations, with click-evoked ABR test assessment being completed in as little as one minute [87]. However, this method relies on the establishment of defined norms of hearing, which are species-specific. Thus, expanding the number of individuals and species for which data are available would provide a more accurate means of interpreting the test. The development of portable, rugged systems with the capacity to test frequency ranges of marine mammals has resulted in more easy-to-administer tests [88,89]. 

### Suggested Welfare Monitoring Measure: Regular Hearing Tests

**Figure 2:**
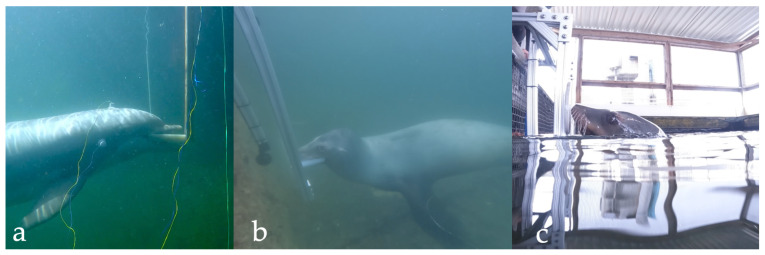
(**a**) A Navy dolphin voluntarily participates in an AEP session in which the animal is wearing electrodes inside soft suction cups that are connected to a computer to record the responses to sound. A Navy sea lion stations on an underwater bite plate during a behavioral hearing test (**b**) and signals detection of a sound by pressing a paddle at the surface (**c**). Photo credit for (**a**), K. Christman; (**b**,**c**), K. Winship.

For facilities caring for marine mammals, investing time and effort to train the population to participate in semi-annual or annual behavioral hearing tests (see Figure 2) would be a straight-forward means to monitor the auditory health of the individuals and detect changes in hearing that could be due to age, illness, or even environmental causes, the latter two reasons negatively impacting welfare and, thus, necessitating intervention and mitigation. At the MMP, hearing assessments are conducted periodically to ascertain dolphins’ ability to perform military tasks, such as intruder detection and object recovery [51,55], and to monitor age-related changes in hearing. A population-wide evaluation of hearing was undertaken at the MMP in 2006, and the authors noted that genetics may play a role in susceptibility to hearing loss. Thus, understanding of an individual’s hearing might impact the prevalence of hearing loss within a breeding program in professional care facilities [51].

Additionally, as some medications are noted to produce ototoxic effects (e.g., aminoglycoside antibiotics, such as amikacin: [72]; gentamicin: [51]), evaluation of hearing following administration of such medications is crucial to monitoring for medication-caused hearing degradation. Given the resistance of some infections to treatment, the utilization of ototoxic antibiotics might be the only viable option [72]; however, monitoring for changes in hearing following treatment is an important consideration when managing the long-term care of the animal post-recovery.

Facilities willing to train a staff member to operate and process data from systems would not only allow for rapid and accurate evaluation of the hearing of the managed population but could enable hearing tests of marine mammals that strand nearby. Often, opportunities to collect data on cryptic species that are only seen when they strand are missed due to the time it takes for trained researchers to travel to the location [88,90]. The MMP and National Marine Mammal Foundation (NMMF) continue to host training opportunities for stranding networks to learn how to operate programs such as EVREST to be used in stranding scenarios, resulting in increased data for common dolphins (*Delphinus delphis*), as well as the first audiograms of Atlantic white-sided dolphins (*Lagenorhynchus acutus*) [90]. Collaborations between facilities and scientists remain viable options to connect animal managers to opportunities to evaluate their marine mammal populations’ hearing. 

## 4. Acoustic Behavior Monitoring

Acoustic welfare-monitoring strategies in terrestrial animals, specifically livestock, have recently seen a rapid increase in their application [91]. Acoustic monitoring of vocalizations [92] and other acoustic signals, such as coughing [93], has been successfully implemented and shown to be useful welfare biomarkers that can lead to reductions in disease spread in herds. In laboratory rats (*Rattus norvegicus*), analysis of ultrasonic vocalizations has been used to monitor instances of positive social affiliation [94] as well as anxiety [95,96]. Technological advancements have allowed for an increase in automated techniques that have made these systems more maintainable. For example, Mao and colleagues [97] reported a 55.88% faster detection speed when using automated monitoring systems for chicken distress calls compared to manual observations. Extensive literature for multiple species exists on the association of vocal behavior and maladaptive behaviors associated with distress, stress, aversion, aggression, and stereotypy [91,93,98,99,100], but the utilization of vocal biomarkers for welfare monitoring has, to our knowledge, yet to be implemented for animals in professional care outside of livestock contexts. 

Numerous studies have identified changes in the acoustic behavior of odontocetes that may be associated with changes in contexts related to their welfare. For instance, Lilly [101] was the first to report on a whistle emitted by a dolphin in distress, possibly to elicit aid from another animal. Similarly, Kuczaj and colleagues [102] observed an ‘incessant’ whistle emission from a wild bottlenose dolphin that could not maintain buoyancy in the water column. The whistle production was associated with helping behavior from conspecifics that provided support to the ill animal at the surface. When a dolphin was in distress, signature whistles were emitted more frequently and with greater intensity [103], and in other studies, a high rate of stereotyped whistle emissions was also reportedly produced by an unwell female dolphin [104]. Capture–release events (i.e., a potentially stressful context) have been associated with a significant increase in the whistle rate and number of whistle loops for dolphins [105,106]. Eskelinen, Richardson, and Tufano [107] found that out-of-water medical procedures were also associated with a significant increase in whistle rate for dolphins, which correlated with an increase in blood cortisol levels. In contrast, in belugas, transport and introduction of another species were associated with a significant and prolonged decrease in vocalization rate [108], suggesting that reductions in acoustic output may also be meaningful. Together, these findings provide a compelling argument that the whistle behavior of cetaceans may provide insights into welfare-related events, such as distress and illness.

### Suggested Welfare Monitoring Measure: Underwater Acoustic Monitoring

**Figure 3:**
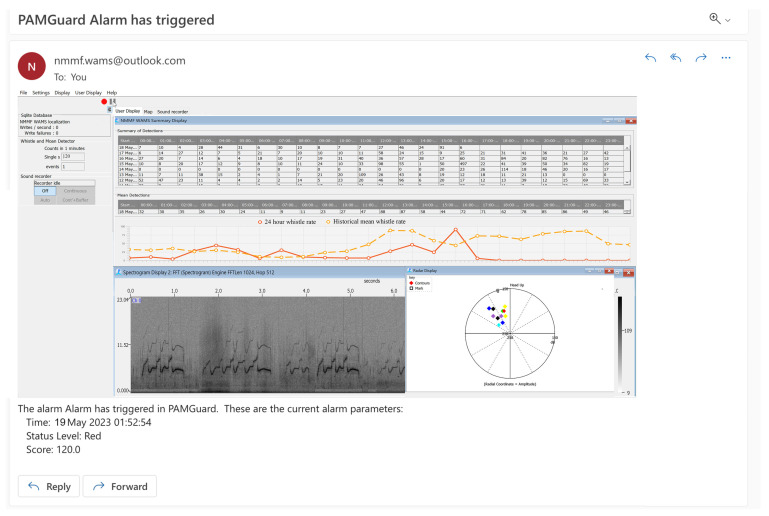
Example of a real-time email alert sent from PAMGuard’s alarm module connected to the NMMF Welfare Acoustic Monitoring System (WAMS). When a full-time hydrophone recording system is installed, WAMS can be utilized to monitor the whistle behavior of the focal group of animals around the clock. The user defines a threshold of number of whistles per minute that would be considered abnormal for that group based on the number of dolphins and typical whistle behavior. If the present whistle rate surpasses that threshold, an email alert is sent to the user-defined email address. Included in the email is a screenshot of the WAMS module (i.e., spectrogram, historical whistle rates, and radar display) from the time the threshold was surpassed. The color boxes on the Radar Display correspond to different whistle contours that have been identified by the program. Additional information on the time of the alarm and the whistle rate recorded are summarized in the body of the email.

Despite the welfare potential of acoustic monitoring for odontocetes in managed care, there has been limited implementation of such systems [108]. However, researchers at the MMP and NMMF have made notable progress by deploying a ‘welfare acoustic monitoring system (WAMS)’ that operates around the clock using a stationary hydrophone array [49]. This system utilizes PAMGuard with a suite of modules, such as the recorder, whistle and moan detector, NMMF WAMS, and alarm module, to create a real-time alert system for abnormal whistle behavior of a group of bottlenose dolphins [48,109]. An example of such an email notification is presented in Figure 3. The audio input is analyzed in real time to identify, localize, and count the whistles emitted by a focal group of dolphins. When the whistle rate surpasses a user-defined threshold within a specified time frame, an email alarm is triggered, complete with the whistle count, a screen grab of the spectrogram, and radar display with localization information, which is immediately sent to a designated email address. Therefore, an animal care lead, night watch, curator, or researcher can receive real-time detailed email alerts when whistle rates reach an abnormal threshold. Abnormally high whistle rates in cetaceans have been reported in multiple contexts that would be of interest to animal care and management, such as distress and dolphin labor [101,102,103,104,110,111].

Although this implementation is a step towards using acoustics to monitor welfare changes in bottlenose dolphins, thresholds of meaningful whistle rates are needed. Arguably, each group of dolphins will have different rates of whistle behavior that are considered normal for that group. Additionally, there is little acoustic data to date on dolphins that are in labor, distress, critically ill, dying, and those that recover from an emergency, such as entanglement or stranding. Collaborations among managed-care facilities and stranding networks to record critically ill dolphins are warranted to help inform these systems and optimize the alarm thresholds to be able to detect these critical changes without a high false-alarm rate. 

## 5. Acoustic Biomarkers of Health

While whistle rate has been the most documented vocal biomarker of distress in bottlenose dolphins, some data suggest that the acoustic features of animal vocalizations may encode biomarkers of health and welfare. Recent technological improvements in acoustic analyses software are allowing for feature analyses to provide even more insights into animal communication systems. For example, goats showed different fundamental frequency and frequency modulation of calls during positively valanced contexts compared to negative ones [112]. Recently, Sadeghi and colleagues [113] found that a model was able to successfully classify what chickens were infected with *Clostridium perfringens* from those that were healthy based solely on the features of their vocalizations. Devi et al. [114] similarly found that five call features were significantly different between the vocalizations of bison calves with and without pneumonia. The authors suggest that bioacoustic features can serve as a non-invasive diagnostic tool for early identification of pneumonia in bison calves. 

### Suggested Welfare Monitoring Measure: Establish Vocal Catalgoue

**Figure 4:**
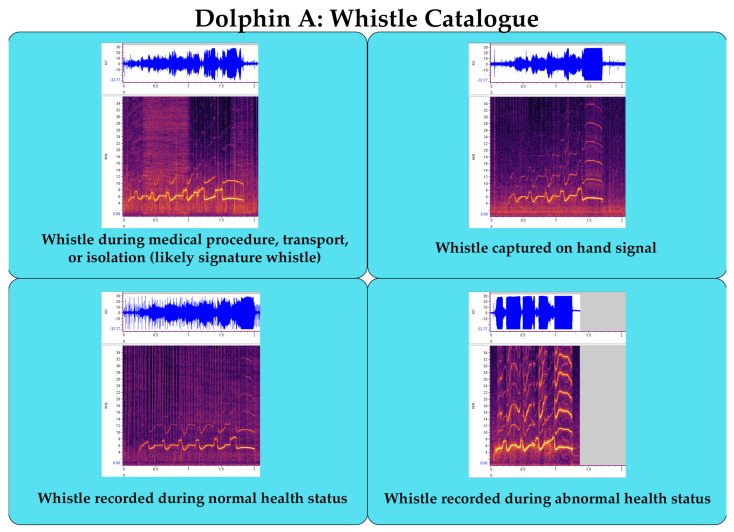
Example of a whistle repertoire for one dolphin. In this example, we depict a spectrogram and waveform (all spectrograms and waveforms were created in RavenPro 1.6; 0–36 kHz on y axis, 8-s time window on the x-axis, Hann window, 2100 window size, 50% overlap, DFT 2048) recorded from a time when Dolphin A was isolated from conspecifics, which is the highest probability of being the dolphin’s signature whistle (distinctive identifier). In the upper-right panel is an example of Dolphin A’s whistle that was recorded when it was produced spontaneously by the dolphin and then paired to a hand signal. The captured whistle is then confirmed to be the same whistle contour as the probable signature whistle in the first panel. The bottom two spectrograms show examples of whistles recorded from the dolphin on a day when it was healthy and a day when it was experiencing an abnormal health status. Whistle catalogues for individual animals during different health conditions can be used to train machine learning classifiers to detect changes in health status from dolphin whistles in the future.

Given these recent successes, it is possible that marine mammal calls may also encode information about health and welfare within the features of the vocalizations. Preliminary research suggests that acoustic biomarkers exist that can be used to classify dolphin health status from their whistles [115]. Early detection of illness is invaluable for improved outcomes and reduced medical costs. Acoustic recordings of marine mammal vocalizations with a known health status (e.g., pulled out of water, transports, medical pools, strandings, rehabilitation centers, etc.) are, therefore, valuable for the future development, application, and generalization of this work. For professionally managed marine mammals, training the individual to provide a vocalization (e.g., a signature whistle by a bottlenose dolphin) and cataloguing the whistle parameters and health status when obtained will help to build a library of examples to train a machine learning model for more accurate predictions of health status (Figure 4). Trainers are able to “capture” an offered signature whistle by reinforcing spontaneous production of the whistle and pairing it with a hand signal or another discriminative stimulus. The trainers can then ask the dolphin to produce the whistle when providing the signal, and the audio can be recorded for analysis.

## 6. Current Acoustic Monitoring Best Practices in Zoos and Aquaria

One of the greatest benefits of acoustic monitoring is the capability to receive information about animals during times that human presence is impossible, dangerous, or difficult (e.g., darkness, late hours, many animals housed together, underwater). While the cost of equipment and a qualified technician for passive acoustic monitoring systems still remains high, potential savings associated with early indication of illness or incident and reductions in medical and caretaker expenses associated should also be considered. Below, we summarize the practices of acoustic welfare monitoring that are currently in place at the MMP (see also Table 1). These tools may be of interest for application at other professionally managed facilities:

1. Annual sound level recordings and supplemental measurements when overt changes are made to the environment (e.g., changes to filtration systems, nearby construction, etc.) to prevent and mitigate potential for hearing impacts [21,46].
2. Hearing tests conducted periodically on the population [51,55]
3. Installation of an underwater acoustic-monitoring system. This system can be utilized for both noise level measurements in the habitats and around-the-clock monitoring (WAMS) of the acoustic behavior of the animals [59]
4. Establishing a vocal catalogue for each animal during different contexts (e.g., positive and negative valence) and health conditions (i.e., normal and abnormal) for machine learning applications of behavioral and health monitoring.

## 7. Future Directions

The continuous acoustic data collected from an acoustic monitoring system allow for enhanced observations in times of interest, such as introductions [108], social interactions, and births [111]. As more research is conducted regarding the whistle rates of various species during different environmental and social contexts, on-board machine learning models can be developed to classify and notify management of behavioral events of interest. The association between burst pulsed sound production and aggressive behavior in bottlenose dolphins has also been well described [116]. Brando et al. [19] identifies rake marks as a physical indicator of aggression. Future research should attempt to correlate burst pulse production and rake mark occurrence to validate burst pulses as an acoustic indicator, leading to a tangible negative health outcome. This is another opportunity for acoustic monitoring to potentially alert animal caretakers to potentially aggressive interactions around the clock, which can be valuable for making management decisions about social groupings, especially overnight. Establishing a better understanding of vocal behavior indicative of positive welfare contexts (e.g., rest, positive social interactions, play) is also important [117].

Understanding how noise might impact cognitive processes is another opportunity for future research. Studies on wild marine mammals suggest that the presence of noise impacts foraging and migration decisions [118,119]. Many dolphin species participate in cooperative feeding strategies [120,121], and there is some evidence of the impairment in this ability in the wild, as well as in controlled cooperative tests [122], due to noise. Navy dolphins experienced a decline in vigilance performance during an echolocation detection task when exposed to vibratory pile-driver noise [123]. The development of a new technological system that provides cognitive enrichment and testing provides a unique opportunity to test, monitor, and compare cognitive skills and performance across animals and species [124]. Utilizing this tool, future research can examine the impact that sound has on additional cognitive processes that can impact life history functions. Additionally, using metrics such as cognitive bias tests in the presence of noise could provide insight on the welfare impacts that various levels of sound have on species [125]. The percentage of correct response, latency to success, or gameplay strategy [126] could be compared between trials in which different levels or types of anthropogenic sound are played. Increased latency, decreased performance, and/or increased use of less efficient strategies could inform the impacts that sound has on marine mammal cognition.

## 8. Conclusions

There is increasing evidence of the impact of anthropogenic sound exposure on marine mammal hearing loss [37,39,42,81], behavioral changes (see [127] for a review), impaired communication [128,129], and reduced foraging efficiency [130,131]. Robust welfare-monitoring protocols for marine mammals in managed care have yet to regularly incorporate acoustic monitoring into their recommendations. We provide evidence that technological advancements have made applied tools for acoustic welfare monitoring accessible. Facilities that are able to invest in these applications through sound level recordings, periodic hearing tests, installation of an underwater acoustic-monitoring system, and/or the establishment of a vocal catalogue will be better able to care for their populations as well as contribute to the continued understanding of the impacts of sound on marine mammal health and welfare in professional care and in the wild.

## Figures and Tables

**Table 1:** Suggested acoustic welfare monitoring practices.

Welfare Monitoring Practice	Timeline	Animal Training Investment	Potential Welfare Benefit	Example
Annual sound level recordings	Annual, with supplemental measurements	None	Mitigation of noise impacts on hearing	A change in filtration system results in an increase in noise in a habitat, which is able to be reduced upon discovery.
Hearing tests (behavioral or AEP)	Periodic (Bi-annual to every three years)	Behavioral: extensive; AEP: can range from none (e.g., during medical procedure) to minimal (e.g., desensitization to equipment) to extensive (e.g., animal trained to participate)	Monitoring for hearing changes related to age, health, and/or environmental impact	A dolphin with age-related hearing loss is transitioned to a visual cue for correct behaviors, resulting in improved performance during training sessions
Underwater Acoustic Monitoring System	Constant	None	Real-time information regarding noise levels and animal acoustic output	Early notification regarding an impending birth is provided due to changes in population whistle rate.
Establish Vocal Catalogue for Individuals	Weekly	Moderate; training animal to produce sounds on cue (e.g., signature whistle in dolphins)	Early detection of behavioral and medical changes within individuals	A machine learning model detects and predicts an abnormal health status, resulting in early identification and treatment and a better health outcome

## Data Availability

Not applicable.
